# Supplementary material for: Leishmania infantum xenodiagnosis from vertically infected dogs reveals significant skin tropism
Source: PLoS Negl Trop Dis. 2021 Oct 6;15(10):e0009366. doi: 10.1371/journal.pntd.0009366 (PMC8523039; doi:10.1371/journal.pntd.0009366)
Supplement: S1 Table — Overview of the age in years, sex, and LeishVet status of xenodiagnosis cohort. (DOCX) [file pntd.0009366.s001.docx]

**S1 Table. Cohort demographics.**

Overview of the age in years, sex, and LeishVet status of xenodiagnosis cohort.
