# Supplementary material for: Leishmania infantum xenodiagnosis from vertically infected dogs reveals significant skin tropism
Source: PLoS Negl Trop Dis. 2021 Oct 6;15(10):e0009366. doi: 10.1371/journal.pntd.0009366 (PMC8523039; doi:10.1371/journal.pntd.0009366)
Supplement: S2 Table — Overview of serum chemistry and blood count findings for dogs in each LeishVet clinical grouping. Bolded values indicate the mean is outside of the normal reference range. (DOCX) [file pntd.0009366.s002.docx]

 **S2 Table. Cohort Bloodwork.**

Overview of serum chemistry and blood count findings for dogs in each LeishVet clinical grouping. Bolded values indicate the mean is outside of the normal reference range. Reference ranges are from IDEXX Laboratories ProCyte Dx or Catalyst Dx machines.
