# Supplementary figures and images for: Leishmania infantum xenodiagnosis from vertically infected dogs reveals significant skin tropism
Source: PLoS Negl Trop Dis. 2021 Oct 6;15(10):e0009366. doi: 10.1371/journal.pntd.0009366 (PMC8523039; doi:10.1371/journal.pntd.0009366)

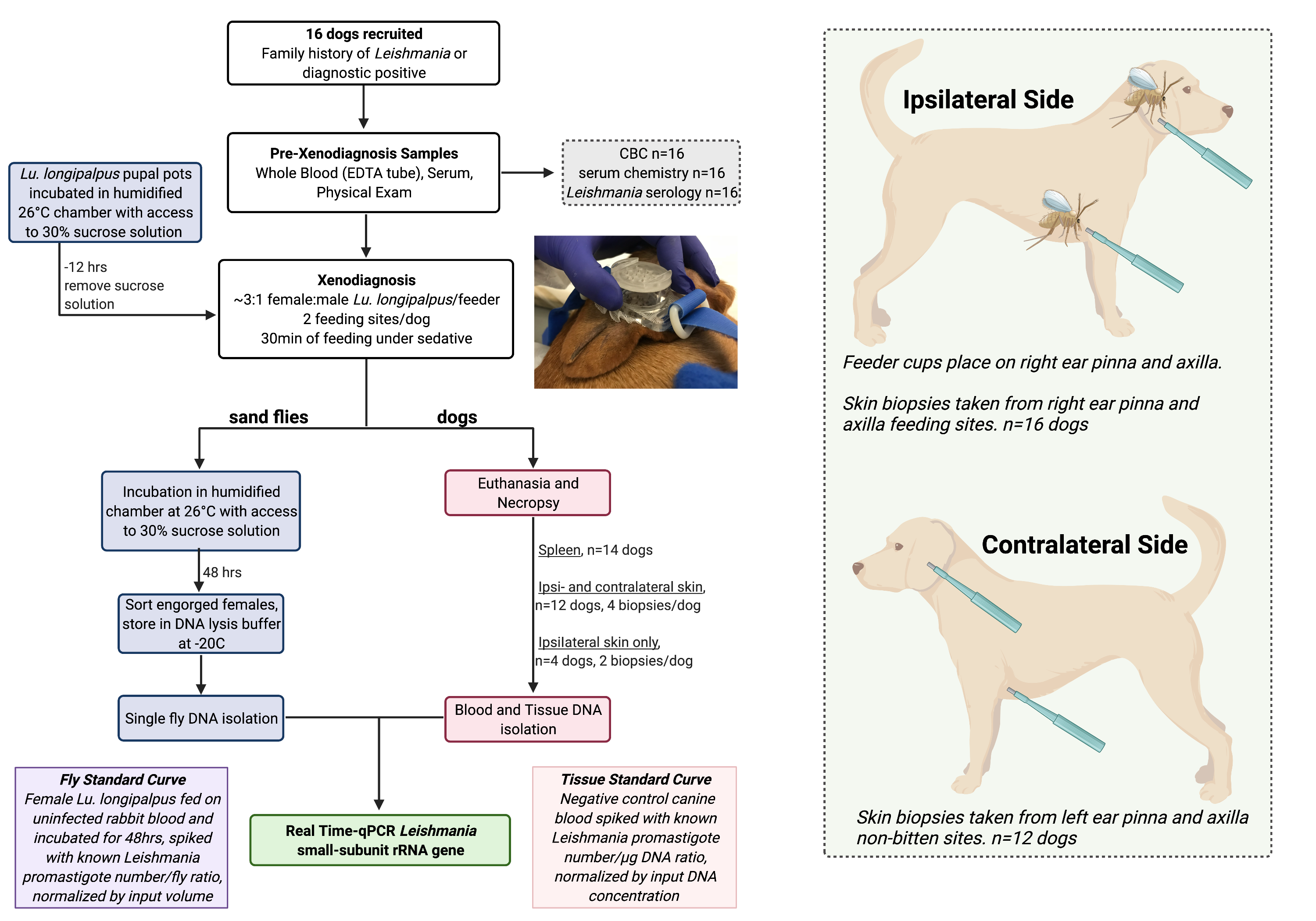

Supplement: S1 Fig — Overview of dogs, sand flies, and sample handling workflow (left). Description of feeding and skin biopsy sites, ipsilateral pertains to side of sand fly feeding and contralateral indicates same location on opposite side where no sand fly feeding occurred. (TIFF) [file pntd.0009366.s003.tiff]

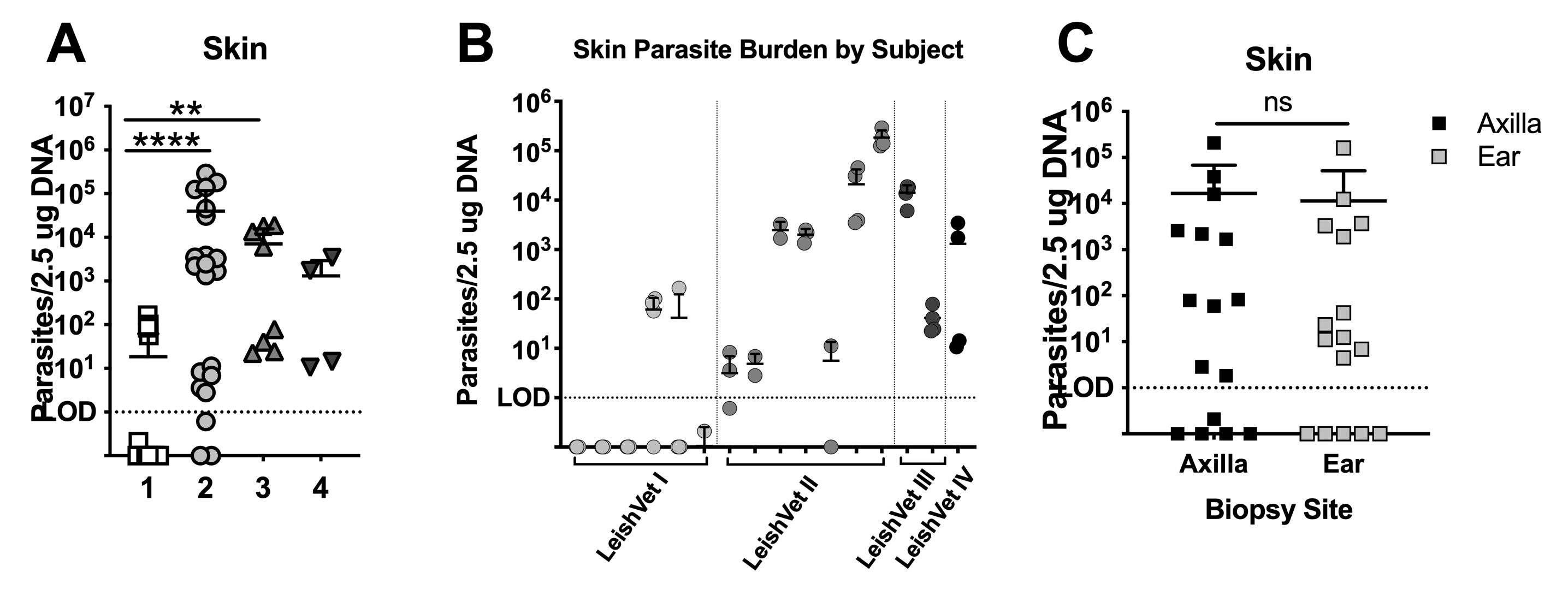

Supplement: S2 Fig — (A-B) Calculated L. infantum parasite burden in skin from dogs at indicated LeishVet clinical stage of disease. (A) Each dot represents one skin biopsy. 2–4 skin biopsies from each dog are shown. Kruskal-Wallis with Dunn’s post-test. (B) Each dot represents one skin biopsy, separated by subject. (C) Calculated L. infantum parasite burden in skin from biopsies collected at either the axillary region or pinna. Each dot represents the average of 1–2 skin biopsies taken at the indicated site from a single dog. Paired Wilcoxon test. Mean and standard deviation are shown for all. (TIFF) [file pntd.0009366.s004.tiff]

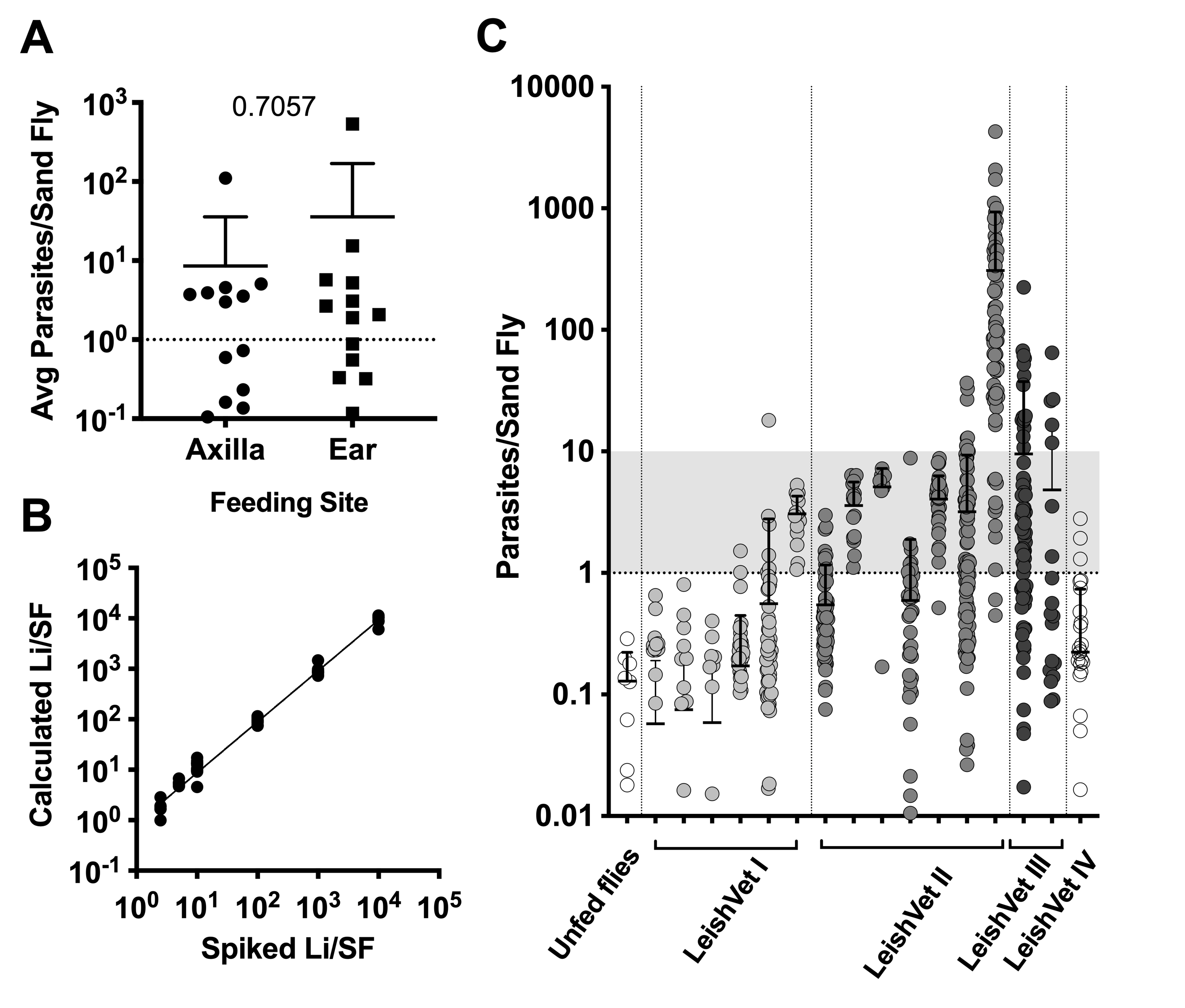

Supplement: S3 Fig — (A) Standard curve derived from Leishmania parasite spiked sand flies Real Time qPCR. (B) Parasite quantification results of all blood-meal containing female sand flies obtained after xenodiagnosis by LeishVet clinical stage. (C) No difference in mean parasite number uptake in blood fed sand flies based on anatomical feeding location via Wilcoxon paired analysis. (TIFF) [file pntd.0009366.s005.tiff]

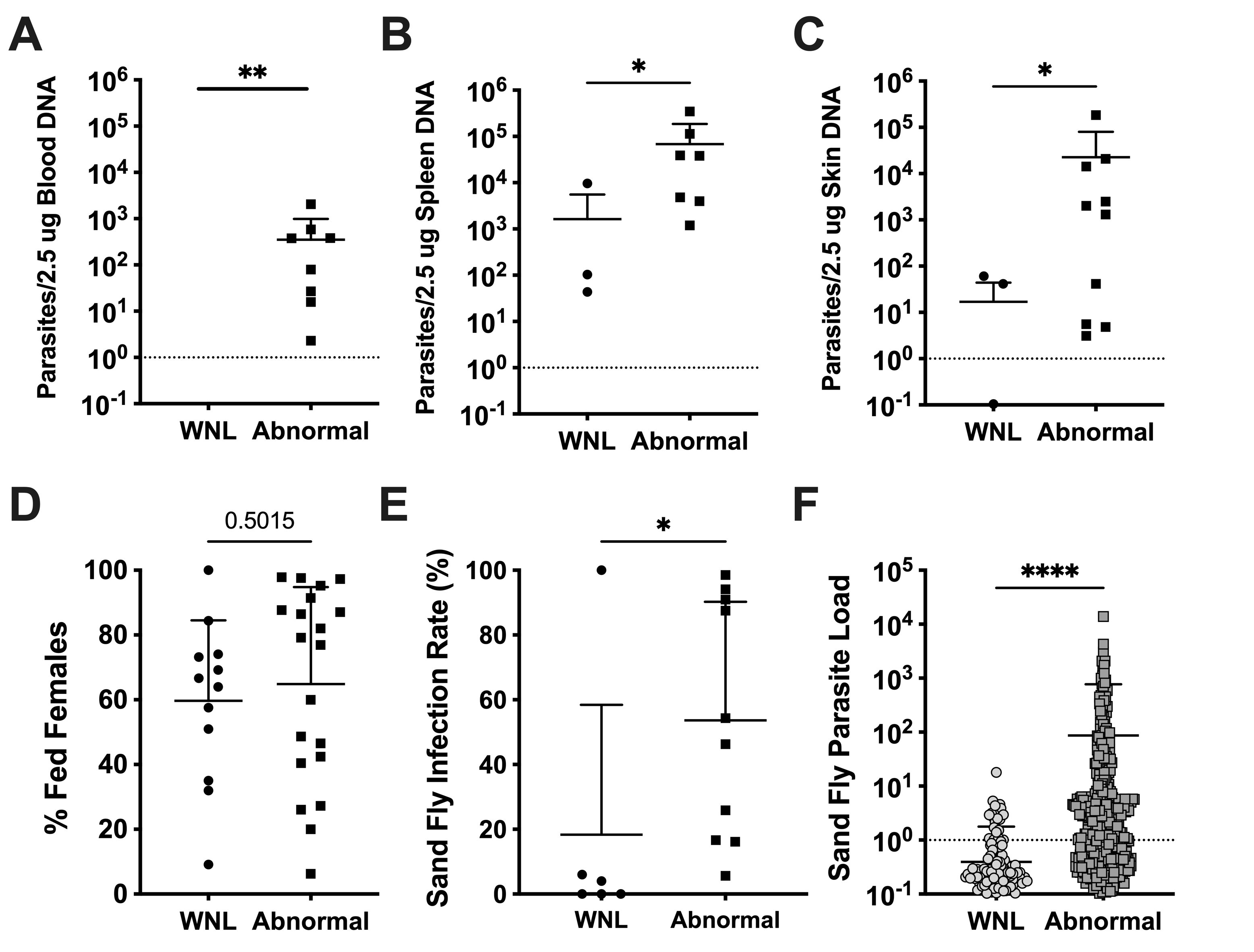

Supplement: S4 Fig — Dogs within normal limits (WNL) were compared against dogs with abnormal complete blood count or serum chemistry clinicopathological findings (Abnormal). Calculated parasite burden in blood (A, n = 16), spleen (B, n = 14), average of 2–4 skin biopsies (C, n = 16). (D) Frequency of female sand flies containing a blood meal after feeding at each site. (E) Frequency of female sand flies containing >1 parasite equivalent 48hrs post-feeding. (F) The calculated parasite burden within sand flies 48hrs post-feeding. (A-F) Mann-Whitney test. *p<0.05; **p<0.01; ****p<0.0001. (TIFF) [file pntd.0009366.s006.tiff]
