# Supplementary material for: Leishmania infantum xenodiagnosis from vertically infected dogs reveals significant skin tropism
Source: PLoS Negl Trop Dis. 2021 Oct 6;15(10):e0009366. doi: 10.1371/journal.pntd.0009366 (PMC8523039; doi:10.1371/journal.pntd.0009366)
Supplement: Attachment — Submitted filename: PLoS NTd comment.docx [file pntd.0009366.s007.docx]

**COMMENTS**

The study by Scorza et al have conducted xenodiagnosis on cohort of dogs to examined infectiousness of vertically infected dogs with *L.infantum* at varying stages of clinical diseases. There are many issues that need to be revised in this current version of the manuscript, to justify the publication in this journal, as follows:

1. Line 91-92 : VL is also caused by L. donovani, and author should make it clear by revising the sentence.
2. Reference 4 is about transplacental transmission in dog but not in humans. Please add appropriate reference.
3. Line 133: How did author confirm the infection?
4. Line: What is the meaning of reader value 10? Is it optical density or percent positives, and how this threshold value change with sample to sample?
5. Line 157 – 160: I guess then approx. 40-50 flies (including males) were exposed at a time during xenodiagnosis, if yes then how did this number come? This is too high number in feeding cups. Please support it with appropriate reference.
6. Line 164: Again, no reference for 30 min exposure to justify best exposure time in dogs.
7. Line 165-166: Why fed flies were incubated for 48 hrs and how this time period was established. As with many other xenodiagnosis studies, researchers use up to 72 hrs flies for outcomes. Please discuss it in detail as this is one of the important factors that can affect the outcomes.
8. Line 165-168: Is there any reason not to perform microscopy for providing direct clinal evidence?
9. Figure 1 do not include negative control (blood, spleen and skin) from healthy dogs and it is difficult to predict threshold without proper control. Author should make it clear in the figure legend. In addition, Figure-1 (A and B) shows the data only 14 dogs instead of 16. I would like to suggest to make schematic flow chart (as supplementary figure) showing all details e.g. recruitment, inclusion and exclusion criteria, samples collection, processing, xenodiagnosis etc. with time periods. This chart is essential to make the study process clear.
10. Figure 1A: how this 2.5 ug DNA was calculated? I guess it should be parasites genome/ml of blood.
11. Figure 1C: Since 2 -4 skin biopsy per dog was sampled as mentioned in line: 230 therefore, please explain the figure about other biopsies data? If it is Figure S2A, then please use the colour dots representing each sample in each category.
12. Line 281- 283: This is what something strange and may be due to false positivity of DNA? Singh et al 2020 (PLoS NTD) has also discussed about this issue with PCR and thus gold standard microscopy xenodiagnosis is needed to confirm such findings. I would like to suggest to perform one set experiment in all these four categories of dog with microscopy xenodiagnosis not only in support of findings but also for direct clinical evidence with live parasite.
13. I don’t understand the need of Figure 3D. Please move it to supplementary or explain the meaning of clinical sign in context to LeishVet Guidelines stages.
14. Fig 3C-F: Please define LOD and how it was calculated?
15. Figure 3A is confusing with regard biopsy number per dogs? Is it cumulative parasite burden or one biopsy only? Please make it clear.
16. Line 358-359: I do not agree with authors as data presented by authors are limited by qPCR. Please discuss it as one of the limitations of study and further studies with microscopy xenodiagnosis are needed to support the findings.
