# Supplementary material for: Leishmania infantum xenodiagnosis from vertically infected dogs reveals significant skin tropism
Source: PLoS Negl Trop Dis. 2021 Oct 6;15(10):e0009366. doi: 10.1371/journal.pntd.0009366 (PMC8523039; doi:10.1371/journal.pntd.0009366)
Supplement: Attachment — Submitted filename: PLoS NTd reviewer comments_Scorza Xeno.docx [file pntd.0009366.s008.docx]

Dear Dr. Hasker,

We have received the reviewers’ comments regarding our manuscript PNTD-D-21-00476 “Leishmania infantum xenodiagnosis from vertically infected dogs reveals significant skin tropism" by Scorza and colleagues submitted to PLOS Neglected Tropical Diseases.

We are thankful for the notes and suggestions raised by the reviewers and the opportunity to improve our manuscript. We have attempted to satisfy all suggested queries to improve the manuscript quality and make it more suitable for publication at PLOS NTD.

Please see point-by-point responses below.

**-------------------**

**Reviewer #1:**

METHODS: The objectives of the study are articulated with a clear testable hypothesis stated.
The study design is appropriate to address the stated objectives.
Statistical analysis used to support conclusions were correct.
There is no concern about meeting ethical or regulatory requirements.

RESULTS: The analysis match the analysis plan.
The results are clearly and completely presented.
Tables and figures in the manuscript and in supporting information are of sufficient quality for clarity.

CONCLUSIONS: The conclusions are supported by the data presented.
The limitations of analysis are clearly described.
The authors discuss in great detail the usefulness of the data to advance our understanding of the topic under study.
Yes, the public health relevance is addressed.

Editorial and Data Presentation Modifications: The subject of this manuscript is within the field of interest for PNTD. It is well written and concise and therefore is suitable for publication in PNTD as Research Article, following some minor revision indicated below.

We thank Reviewer #1 for the kind comments regarding our manuscript. We have addressed the changes requested in the manuscript text as suggested.

1.  There is no doubt that the skin parasite load seems a better predictor than peripheral blood in canine leishmaniasis and probably in PKDL patients. However, in the case of human VL caused by L. infantum this is not so clear. Although the skin seems to play some role in immunosupressed L. donovani-infected patients it must also be taken into account that venous blood also is an important source of parasites for sand flies in the case of HIV / L. infantum coinfection. Furthermore, in the case of the human visceral leishmaniasis caused by Leishmania infantum the proportion of patients harboring parasites in their healthy skin is relatively low and such parasitism, although unusual, may be a source of infection for phlebotomine sand flies (Moura CRLP Costa CHN, Moura RD, Braga ARF, Silva VC, Costa DL. Cutaneous parasitism in patients with American visceral leishmaniasis in an endemic área. Revista da Sociedade Brasileira de Medicina Tropical. 2020; 53:e20190446.
https://doi.org/10.1590/0037-8682-0446-2019). Therefore, it is very important to definitely clarify the real role of parasite burden in the blood and skin of immunocompetent and immunosuppressed individuals in the transmission of human VL.

We have added in the discussion section a paragraph regarding active human VL skin parasite burdens and that high levels of parasitemia observed in HIV coinfected patients may increase the ability of blood to serve as source of parasites for sand flies. Thank you for pointing out this gap and for providing the reference, we agree discussion of human skin vs blood contributions will be a positive addition to this manuscript.

2.  Lines 330-339. I think that in the discussion addressed in this paragraph it would be very enriching also discuss the work of Mondal et al, 2018 (Mondal D, Bern C, Ghosh D, Rashid M, Molina R, Chowdhury R, Nath R, Ghosh P, Chapman LAC, Alim A, Bilbe G, Alvar J. Quantifying the infectiousness of post-kala-azar dermal leishmaniasis toward sand flies. Clinical Infectious Diseases. 2018; ciy891. https://doi.org/10.1093/cid/ciy891) jointly with the work of Singh et al, 2021 (<https://doi.org/10.1016/S2666-5247(20)30166-X>).

Thank you for realizing we missed this citation; we accidentally cited a different paper from Mondal et al. 2010. We agree it is highly relevant and have replaced the citation about this study.

3.  Why the study was not able to capture whether all infected sand flies would go on to develop high levels of infection and virulent metacyclic promastigotes?

Our institution where xenodiagnosis was performed does not allow sand fly insectary facilities. We collaborated with insectaries at NIAID and University of Madison to rear our sand flies and received them as emerging adults from pupal pots. Therefore, we had to limit incubation of sand flies in our lab post-feeding on *Leishmania* infected animals per our institutional Biosafety committee requirements. We were limited to incubation of sand flies post-xenodiagnosis to 48hrs, to minimize the risk of transmission to humans in the case of an escaped fly.

As our research question was about transmission to sand flies, we were able to achieve our experimental results/goals in this time frame.

We have added the following clarification to the methods section regarding post-feeding: “Due to institutional requirements, 48hrs was the maximum allowable incubation time post-feeding on infected dogs to minimize risk of infection to staff.”

Summary and General Comments: This is an excellent work well-designed and conducted. The authors show in this study that canine vertical transmission of leishmaniasis should be taken into consideration in surveillance and prevention strategies. Furthermore the authors corroborate that parasite burden of dog skin can be a strong indicator of outward transmission potential.

With this paper one more piece is provided to the puzzle of the canine leishmaniasis epidemiology.

**Reviewer #2:**

Summary and General Comments:

There are some issues that the authors must consider. The manuscript at this moment should not be recommended or needs major revisions before to evaluate its publication.

Major comments

I suggest review the experimental design used to classify CanL disease. The authors described that CanL classification used in the study was according to Leishvet guideline. However, the stages defined in this study as "subclinical (stage 1), mild (stage 2), moderate (stage 3), or severe disease (stage 4)" are not according to the LeishVet reference. The Leishvet guideline establishes CanL disease as "stage I (mild disease), stage II (moderate disease), stage III (severe disease) and stage IV (very severe disease)". If the author adapted the CanL classification, the used criteria should be described.

Thank you for noticing this discrepancy. We did use the LeishVet scale as described by the guideline authors. We have corrected two instances of mislabeled LeishVet stage descriptions as indicated by LeishVet guidelines.

Since small sample was evaluated in this study, I suggest the authors consider the reclassification of dogs according to "no clinicopathological alterations versus with clinicopathological alterations".

We have added a supplemental figure depicting the comparison of tissue parasite loads and xenodiagnosis results separating dogs by those without any clinicopathological abnormalities (LeishVet stage 1, n=6) vs those with clinicopathological alterations (LeishVet stage 2-4, n=10). We have added text to the results section and discussion highlighting these findings.

The authors describe that skin parasite burden is more important than dog´s clinical status. I suggest to reword the sentences with these statements, since LeishVet stage 3 and 4 had few dogs, 2 and 1, respectively. Their emphasized does not appear appropriate.

We have reworded the statement.

In Material and methods section should provide more information about dogs sampling. What is their origin? How was performed the follow up of these dogs since birth? It is not clear if after the birth, the dogs of this study have never been exposed to areas with potential risk for L. infantum infection transmitted by the vector.

The dogs used in this study were obtained from a U.S. from a cohort where vector transmission of *L. infantum* was ruled out based on the high prevalence among the cohort but virtually absent serologically positive other pet dogs, shelter dogs, and wild canids in the local proximity to the study population. We have added a sentence indicating these dogs are from the U.S. and some references describing the cohort. These dogs have no travel history outside of the US, and in more than half of these dogs n=10, we have performed yearly active surveillance of their kennel since birth.

“Canine subjects were obtained from a cohort of U.S. dogs where vertically transmitted *L. infantum* is enzootic.”

- https://doi.org/10.3201/eid1203.050811
- DOI: 10.1016/j.vprsr.2017.08.011
- https://doi.org/10.1371/journal.pntd.0001019

In figure 1, is not clear how statistical analysis was performed. How was performed each group comparison? For example, parasite burden in blood was significantly higher compared with what? Line 215-216 in the statement L. infantum parasite burden in (A) blood (p=0.004; n=16), (B) spleen (p=0.153; 216 n=14), or (C) skin (p=0.072; n=16), these P value refers to comparison between blood and spleen or skin..., please clarify. I suggest include in figure 1 another graph to show parasite burden per tissue without CanL stages.

The p-values reported in the Figure 1 legend were calculated an Kruskal-Wallis (non-parametric ANOVA test) without multiple comparisons, therefore a significant p-value indicates significant variation between the medians in the different groups. This result means that median tissue burden is significantly influenced based on LeishVet status overall but does not tell which LeishVet stages drive the difference. Since it is a little confusing, we have removed the results of this test. We have also added the tissue comparison panel graph to Figure 1 as requested.

Minor comments

I suggest using the term "canine leishmaniasis (CanL)” when authors refer the dog disease.
Line 97.

As suggested, we have adjusted the reference from “humans and dogs with VL” to “both humans and dogs infected with *L. infantum”.*

References (21, 22) are not correctly numbered in the order that they appear in the text.

We have updated the citations and corrected this.

Line 127 change ‘confirms’ to ‘suggests’

We have made the change as suggested.

Line 287 change ‘subclinical disease’ to subclinical infection

We have made the change as suggested.

In table S1, I suggest to provide age class frequency (young, adult or elderly). Exclude ‘mean (SD)’. Also, I suggest include information on breed.

We have updated Table S1 as requested.

In table S2, I suggest to include the reference source which was take out the value normal range of the laboratory data.

We have added a sentence to the Table S2 legend indicating the source of normal ranges: “Reference ranges are from IDEXX Laboratories ProCyte Dx or Catalyst Dx machines.”

Lines 172-173 the statement “Skin biopsies were taken from axillary and pinnal sand fly feeding sites as well as contralateral”, I suggest to provide more information on number fragment tissues were obtained from each skin region.

We have added the indicated information in an additional supplemental workflow figure (Figure S1) which should help clarify skin biopsy locations along with the text.

Line 292-293. On the statement “the age range of dogs with the highest average parasite uptake per sand fly in this cohort was 3-4 years old”, what is hypothesis for this finding?

Our hypothesis is based on previous findings that IFNg itself stimulates parasite growth, and dogs in Leishvet stage 2-3 have been shown to have good T helper 1 type immunity which would lead to outgrowth of parasites. We have found that the average age of death in this cohort is 6 years old, so beyond this controlled stage dogs progress and are dying.

Cell Host Microbe. 2020 May 13;27(5):752-768.e7. doi: 10.1016/j.chom.2020.03.011. Epub 2020 Apr 15.

Line 305-306. In the statement “Parasitemia is known to correlate with Leishmania parasite transmission to sand flies”, provide reference.

We have added three references which found parasitemia correlated with xenodiagnosis at the indicated statement.

Line 360-362. The statement “Moderately diseased dogs (LeishVet levels 2-3) were the most infectious to *Lu. longipalpis* sand flies compared to dogs with mild (LeishVet level 1) or severe disease (LeishVet level 4)” is confused. It is not according to CanL classification used by authors (line 151-152).

We have corrected the mislabeling of the LeishVet stages according to the LeishVet guidelines authors description of each stage.

Line 394-396 statement “Our data supports skin parasite load as a surrogate marker of dogs with L. infantum transmission potential from both horizontally and vertically infected dogs”, I suggest exxclude “horizontally”, since this study not compared parasite skin load from horizontally infected dogs.

We have updated the statement as suggested: “In agreement with studies in vector infected dog cohorts, our data supports skin parasite load as a surrogate marker of dogs with *L. infantum* transmission potential vertically infected dogs.”

**Reviewer #3:**

METHODS:

1.    Line 91-92 : VL is also caused by L. donovani, and author should make it clear by revising the sentence.

We have added that both *L. infantum* and *L. donovani* cause VL.

2.    Reference 4 is about transplacental transmission in dog but not in humans. Please add appropriate reference.

We have added 4 references containing reports of human vertical transmission.

Mescouto-Borges et al.

Galindo-Sevilla et al.

Meinecke et al.

Boehme et al.

3.    Line 133: How did author confirm the infection?

We cultured *Leishmania* parasites from splenic tissue from 8 dogs in order to verify live parasites. Of the 8 dogs that did not undergo culture, 6/8 were diagnostically positive by qPCR in one or more tissues and 6/8 were diagnostically positive by serology using the DPP®CVL test.

Overall, 13/16 dogs were diagnostically positive by qPCR in one or more tissues and 13/16 dogs were serologically positive using the DPP®CVL test.

There was only one dog that did not have a positive diagnostic test or parasite culture performed. This dog did however have both their dam and full siblings test positive by qPCR of whole blood by our laboratory in previous surveillance testing. We know from years of surveillance testing multiple generations of dogs from this cohort that this is highly indicative that this dog was exposed to parasites *in utero* and harbored an infection below the detectable limit.

4.    Line: What is the meaning of reader value 10? Is it optical density or percent positives, and how this threshold value change with sample to sample?

The ChemBios electronic micro reader is a proprietary digital reader provided to us by the company along with the DPP ®CVL serological tests. It calculates a reader value based on the reflectance intensities of the test band and control band, and uses an assay specific algorithm to report band intensity in arbitrary units. The cutoff of 10 is set by the algorithm and manufacturer.

5.    Line 157 – 160: I guess then approx. 40-50 flies (including males) were exposed at a time during xenodiagnosis, if yes then how did this number come? This is too high number in feeding cups. Please support it with appropriate reference.

The inclusion of the 3:1 ratio of females to males and the number of flies per container were decided based on the recommendation of our collaborator and co-author Dr. Shaden Kamhawi who has extensive experience with leishmaniasis, phlebotomine sand fly rearing, and xenodiagnosis with dogs, mice, and hamsters. Here are some references which have utilized a similar number of flies and feeding time successfully which we have added citations for in the sand fly and xenodiagnosis section of the methods:

- https://doi.org/10.1016/S2666-5247(20)30166-X
- DOI: 10.1038/s41467-017-00103-8
- https://doi.org/10.1016/j.vetpar.2016.10.004

6.    Line 164: Again, no reference for 30 min exposure to justify best exposure time in dogs.

Similar to the previous comment, 30 minutes was not determined empirically by us as the best exposure time, but is known from previous publications and personal experience of collaborators to be sufficient for sand fly feeding. We have added the citations after the indicated sentence as well.

7.    Line 165-166: Why fed flies were incubated for 48 hrs and how this time period was established. As with many other xenodiagnosis studies, researchers use up to 72 hrs flies for outcomes. Please discuss it in detail as this is one of the important factors that can affect the outcomes.

Our institution where xenodiagnosis was performed does not have official sand fly insectary facilities. We collaborated with the insectaries at NIAID and University of Madison to rear our sand flies and only received them as emerging adults. Therefore, we had to incubate sand flies in our lab post-feeding on Leishmania infected animals. Our Biosafety committee approval for these experiment limited the amount of time we could harbor infected sand flies post-xenodiagnosis to 48hrs, to minimize the risk of transmission to humans in the case of an escaped fly.

As our research question was about transmission to sand flies, we were able to achieve our experimental results/goals in this time.

We have added the following clarification to the methods section regarding post-feeding: “Due to institutional requirements, 48hrs was the maximum allowable incubation time post-feeding on infected dogs to minimize risk of infection to staff.”

8.    Line 165-168: Is there any reason not to perform microscopy for providing direct clinal evidence?

Microscopy is not as sensitive as RT-qPCR for detecting small amounts of parasites. As we performed xenodiagnosis on dogs across the infection spectrum including, dogs with very low parasite burdens, and only cultured fed sand flies for 48hrs, we did not feel microscopy would be a reliable method for quantifying ingested parasites within sand flies. Many studies have used qPCR for quantifying ingested parasites after xenodiagnosis. Additionally, Singh et al. 2021 recently reported in The Lancet that when using both methods after *P. papatasi* xenodiagnosis, all sand flies positive for xenodiagnosis by microscopy were also positive by qPCR, showing a high degree of agreement between the two methods.

RESULTS:

9.     Figure 1 do not include negative control (blood, spleen and skin) from healthy dogs and it is difficult to predict threshold without proper control. Author should make it clear in the figure legend. In addition, Figure-1 (A and B) shows the data only 14 dogs instead of 16. I would like to suggest to make schematic flow chart (as supplementary figure) showing all details e.g. recruitment, inclusion and exclusion criteria, samples collection, processing, xenodiagnosis etc. with time periods. This chart is essential to make the study process clear.

We were unable to obtain healthy control spleen or skin canine samples, but used input normalized, non-*Leishmania* exposed, negative control canine blood DNA as a negative control. Uninfected canine blood does not amplify using our *Leishmania*-specific primers so would be assigned a calculated parasite load of zero/below LOD.

Figure panel 1A and 1C contains data from all 16 dogs. Figure panel 1B contains data from 14 dogs.

We agree that a chart always helps to clarify experiment workflow. We have made a biorender flow chart depicting the steps of the experiment, location of biopsies, and types of samples. We have added this flow chart as a supplemental figure.

10.    Figure 1A: how this 2.5 ug DNA was calculated? I guess it should be parasites genome/ml of blood.

DNA isolated from skin and spleen tissue samples was normalized to include an equal amount of input DNA in each RT-qPCR reaction. When only assaying blood parasite load, as the reviewer suggested, we usually would calculate as parasite genome equivalents per mL of blood, however to be able to directly compare calculated parasite burdens between blood and tissues and perform paired statistics, we normalized the blood DNA input to 2.5ug as well.

11.     Figure 1C: Since 2 -4 skin biopsy per dog was sampled as mentioned in line: 230 therefore, please explain the figure about other biopsies data? If it is Figure S2A, then please use the colour dots representing each sample in each category.

The reviewer is correct that Figure 1C shows the average skin parasite burden from 2 (n=4) or 4 (n=12) skin biopsy locations/dog. Figure S2A and S2B show the skin parasite burdens for individual biopsies separated by LeishVet stage (Figure S2A) or by individual canine subject (Figure S2B). We have made each LeishVet category different shades of gray and separated the different stage categories with a dotted vertical line in Figure S2B. We hope this is sufficient to visualize the variation in skin parasite load between biopsies taken from a single subject.

12.    Line 281- 283: This is what something strange and may be due to false positivity of DNA? Singh et al 2020 (PLoS NTD) has also discussed about this issue with PCR and thus gold standard microscopy xenodiagnosis is needed to confirm such findings. I would like to suggest to perform one set experiment in all these four categories of dog with microscopy xenodiagnosis not only in support of findings but also for direct clinical evidence with live parasite.

We believe the LeishVet stage 2 group in our study led to the most infected sand flies because of some dogs in that group had particularly high skin and blood parasite burdens, leading to efficient uptake by the vector, and not due to an issue with qPCR. Although each dog was assigned a LeishVet stage at the time of xenodiagnosis, the disease is dynamic, and a dog may fluctuate with mild/moderate disease before progressing to severe disease. These results are similar to those of Courtenay et al (2014) from sand fly transmitted *L. infantum*-infected dogs in Brazil.

In the Singh et al. Plos NTD study, Dr. Singh highlighted that in another study of dogs with CanL due to *L. infantum* by Laurenti et al. 2013, asymptomatic dogs were significantly more infectious to sand flies compared to symptomatic dogs. This study used microscopy to identify infected sand flies which were confirmed by PCR. We did not see any indication in Singh et al. 2020 about potential false positive PCR concerns. We speculate that there is great potential for false negative results and observer bias when using microscopy.

13.     I don’t understand the need of Figure 3D. Please move it to supplementary or explain the meaning of clinical sign in context to LeishVet Guidelines stages.

In figure 3D, we sort the sand fly parasite uptake results by the number clinical signs observable by physical exam a dog showed on the day of xenodiagnosis. This is distinct from the LeishVet stage of disease, which is more robust and includes clinical laboratory test findings. Many studies of canine VL only use physical clinical signs to classify clinical status of the dog as asymptomatic or symptomatic (sometimes broken down into oligo- vs poly-symptomatic subcategories). In order for researchers to rigorously compare our results to previously published xenodiagnosis results based on a specific and clear clinical scoring system, we thought it was important to include this categorization.

We have added descriptions of the clinical signs of leishmaniosis included in physical exams of dogs undergoing xenodiagnosis and updated the results section text to be more clear.

14.    Fig 3C-F: Please define LOD and how it was calculated?

LOD is limit of detection, we used it to signify 1 parasite equivalent as a cut-off for ‘real’ or ‘acceptable’ parasite presence, as anything less than one cannot be a whole parasite.

15.    Figure 3A is confusing with regard biopsy number per dogs? Is it cumulative parasite burden or one biopsy only? Please make it clear.

We believe the reviewer is referring to figure 4A. For this panel, skin parasite burden was averaged between the two biopsies collected only from the ipsilateral sand fly feeding sites. Sand fly parasite uptake is correlated to skin parasite load from the matched sand fly feeding locations. We have added a line to the Figure 4 legend to clarify this.

CONCLUSIONS:

16.    Line 358-359: I do not agree with authors as data presented by authors are limited by qPCR. Please discuss it as one of the limitations of study and further studies with microscopy xenodiagnosis are needed to support the findings.

We understand that microscopy and qPCR each have pros and cons. We have included a statement in the discussion identifying the limitation:

“Although we quantified parasite uptake after xenodiagnosis with a high degree of sensitivity utilizing qPCR, a limitation of this study is that parasite viability in the sand fly midgut was not verified by microscopy, and therefore we cannot assume all parasites survived ingestion.”

Summary and General Comments: The study by Scorza et al have conducted xenodiagnosis on cohort of dogs to examined infectiousness of vertically infected dogs with L. infantum at varying stages of clinical diseases.
